# c-kit Positive Cardiac Outgrowth Cells Demonstrate Better Ability for Cardiac Recovery Against Ischemic Myopathy

**DOI:** 10.4172/2157-7633.1000402

**Published:** 2017-10-13

**Authors:** Chuan Li, Satoshi Matsushita, Zhengqing Li, Jianjun Guan, Atsushi Amano

**Affiliations:** 1 Department of Cardiovascular Surgery, Juntendo University Faculty of Medicine, Tokyo, Japan; 2 Department of Materials Science and Engineering, Ohio State University, Columbus, USA

**Keywords:** Stem cell therapy, Angiogenesis, Anti-apoptosis, Heart regeneration, Myocardial infarction, Cardiac stem cells

## Abstract

**Objective:**

Resident cardiac stem cells are expected to be a therapeutic option for patients who suffer from severe heart failure. However, uncertainty remains over whether sorting cells for c-kit, a stem cell marker, improves therapeutic outcomes.

**Materials and methods:**

Cardiac outgrowth cells cultured from explants of rat heart atrium were sorted according to their positivity (+) or negativity (−) for c-kit. These cells were exposed to hypoxia for 3 d, and subsequently harvested for mRNA expression measurement. The cell medium was also collected to assess cytokine secretion. To test for a functional benefit in animals, myocardial infarction (MI) was induced in rats, and c-kit+ or c-kit− cells were injected. The left ventricular ejection fraction (LVEF) was measured for up to 4 weeks, after which the heart was harvested for biological and histological analyses.

**Results and conclusion:**

Expression of the angiogenesis-related genes, VEGF and ANGPTL2, was significantly higher in c-kit+ cells after 3 d of hypoxic culture, although we found no such difference prior to hypoxia. Secretion of VEGF and ANGPTL2 was greater in the c-kit+ group than in the c-kit− group, while hypoxia tended to increase cytokine expression in both groups. In addition, IGF-1 was significantly increased in the c-kit+ group, consistent with the relatively low expression of cleaved-caspase 3 revealed by western blot assay, and the relatively low count of apoptotic cells revealed by histochemical analysis. Administration of c-kit+cells into the MI heart improved the LVEF and increased neovascularization. These results indicate that c-kit+cells may be useful in cardiac stem cell therapy.

## Introduction

Despite improvements in medical treatment for heart diseases, many patients still suffer from heart failure [1]. The final therapy for end-stage heart failure is heart transplantation; however, the number of donors is limited. Since the most common disease that develops into cardiac failure is Myocardial Infarction (MI), it has been anticipated that the number of patients with severe heart failure will grow with the number of MI patients. Cardiac regeneration featuring stem cell treatment is a novel therapeutic approach that is expected to prevent severe heart failure or remediate its consequences [2]. Most efforts at cardiac regenerative treatment have yet to demonstrate consistent efficacy [3].

Recently, resident Cardiac Stem Cells (CSCs) were discovered, and the method to culture them was established [4]. Around these years, the researchers distinguish the CSCs by using different protein markers, which show different specialties. The c-kit, Sca-1 (Stem cells antigen-1), and islet-1 proteins are all considered markers of CSCs [5–7]. Among these CSC markers, c-kit is the most commonly used [8–11] because c-kit positive (c-kit+) cells have been shown to differentiate into cardiac cells including cardiomyocytes, vascular smooth muscle cells, and endothelial cells [8,42]. These cells can reduce the size of MI scars and maintain the left ventricle ejection function (LVEF) *in vivo* [9], and are considered the primary factors driving myocardium regeneration following myocardial infarction [12]. Furthermore, the c-kit+ cell therapy has been extended to clinical trials that utilized autologous c-kit+ cells to cure low LVEF heart [13].

In contrast, favorable recovery of cardiac function has also been demonstrated by other trials using non-cardiac stem cells, myoblast cells [14], or endothelial progenitor cells co-cultured with fibroblasts [15]. In addition, another human clinical trial using autologous cardiosphere-derived cells (CDCs), which contain heterogeneous proportion with 5–10% of c-kit+ cells and dominant population of non-positive cells, reports that the patients receiving the intracoronary infusion of CDCs showed a better recovery of the scar size than the control group [16,17]. Furthermore, previous report demonstrated that c-kit+ cells minimally contribute to the cardiomyocytes in the heart [18]. The benefits of a sorted c-kit+ cell treatment versus those of a complex cell treatment have yet to be fully understood [18]. We therefore used *in vitro* experiments and an *in vivo* rat heart model of MI to directly compare c-kit− cells with c-kit+ cells.

## Materials and Methods

### Animal care

Experimental animals were treated in compliance with the institutional guidelines for animal experimentation of the Institutional Animal Care and Usage Committee (IACUC) of Juntendo University, School of Medicine. All experimental procedures were approved by IACUC of Juntendo University.

### Preparation of the cells

The cells were cultured from atrium of the green fluorescent protein (GFP)-expressing male Sprague-Dawley rat (SD-Tg[CAG-EGFP]; Sankyo Lab, Tokyo, Japan) hearts. Under anesthesia, the heart was dissected and perfused with phosphate buffered saline (PBS; Wako, Tokyo, Japan) containing heparin sodium (Mochida Pharma, Tokyo, Japan) to wash out the blood. The atrium of the heart was next collected, cut into small pieces (less than 1 mm), and digested with 0.05% trypsin-ethylenediaminetetraacetic acid (EDTA; Sigma-Aldrich, Tokyo, Japan) for 9 min. These pieces were plated onto fibronectin-coated dishes (BD Biosciences, Tokyo, Japan) in Iscove’s modified Eagle’s medium (Life Technologies, Tokyo, Japan) supplemented with 20% fetal bovine serum (Thermo Scientific, Yokohama, Japan), 1% penicillin-streptomycin (Life Technologies, Tokyo, Japan). Two weeks later, the adherent outgrowth cells grew radially and were harvested to culture until second passage to expand the number of the cells.

### Cell sorting

When the cells were confluent, we conducted fluorescence activated cell sorting (FACS), using phycoerythrin (PE)-conjugated anti-c-kit antibody and isotype control (Bioss, Boston, MA, USA), with a flow cytometer (Beckman Coulter, Moflo Astrios EQs, Tokyo, Japan). Each of positive or negative for c-kit cells went on culturing separately. After 2 weeks, the cells were harvested for injection or seeding into 6-well plates (5.0 × 10^4^ cells/well) for *in vitro* study.

### Hypoxic culture environment

For hypoxic culture, the 6-well plates were placed into the multi-gas incubator (CO_2_/Multi-gas incubator Water Jacket, Astec, Tokyo, Japan) in the condition temperature 37°C, 3% of O_2_, 5% of CO_2_ with 1.5 mL of medium per well. On the day before placing into the hypoxic incubator (day 0), and 3 d after hypoxic culture (day 3), the cells were harvested to perform further experiment. The medium of culture cells was changed every day. Before harvesting the cells, the medium was collected for enzyme-linked immunosorbent assay (ELISA) at the same time points (day 0 and day 3).

### RT-PCR (Reverse transcription polymerase chain reaction)

A portion of the harvested cells was prepared for RT-PCR analysis. The RNA was extracted using an RNeasy Mini Kit (Qiagen, Tokyo, Japan), and subsequently reverse-transcribed using an iScript cDNA Synthesis Kit (Bio-Rad, Tokyo, Japan), to generate cDNA. The PCR reaction and analysis were carried out using an HT-7700 thermocycler (Life Technologies, Tokyo, Japan) with the SYBR-green kit (Life Technologies, Tokyo, Japan). Primers were designed to detect vascular endothelial growth factor (VEGF), angiopoietin-like 2 (ANGPTL2), and insulin-like growth factor-1 (IGF-1). The relative abundance of each target gene was determined by normalizing the gene-specific mRNA levels to that of an internal control (GAPDH).

### ELISA

The collected culture medium was analyzed using ELISA kits for VEGF, ANGPTL2 (Cloud-Clone Corp, Houston, TX, USA), and IGF-1 (R&D Systems, Tokyo, Japan). The absorbance of each sample was measured at 450 nm using Spectra max 340 PC (Molecular Devices, Sunnyvale, CA, USA). Cytokine levels were determined from standard curves and expressed in pg/mL.

### Rat model of myocardial infarction

A myocardial infarction model was prepared using eight-week-old male SD rats (weight: 240–280 g). After anesthetization with an intra-peritoneal injection of sodium pentobarbital (Somnopentyl, 30 mg/kg; Kyoritsu Seiyaku, Tokyo, Japan), the rat was intubated with a 14-gauge angiocatheter (Terumo, Tokyo, Japan). Artificial ventilation (volume of 4.5 mL and respiration rate of 70 breaths/min) was next connected to the gauge. A lateral incision was made to approach the heart, followed by ligation of the left coronary artery with a 7-0 monofilament suture (Prolene; Ethicon Japan, Tokyo, Japan). Immediately after ligation, 2.0 × 10^5^ c-kit+ or c-kit− cells were injected into the peri-infarct area, using a 26-gauge needle. To maximize the effect of the cells, they were injected in 70 µL of a thermosensitive and biodegradable hydrogel. This gel has been described previously [19,20]. For a control group, we injected the same volume of the hydrogel with no cells.

### Cardiac function

Cardiac function was evaluated by micro computed tomography (mCT, Latheta LCT-200; Hitachi-Aloka Medical, Tokyo, Japan) at the surgery day (baseline) and 1 and 4 weeks after the surgery. The rats were anesthetized using the method described above, and body weight was measured to determine the volume of the contrast iodized medium (Bystage 370; Teva Pharma, Tokyo, Japan). The procedure for the evaluation of heart function has been described previously [21]. After 4 weeks of heart function measurement, the heart was taken out and prepared for western blot and histological experiment.

### Histological analysis

The section of the heart encompassing the peri-infarction area, between the apex and ligation place, was immersed in 4% paraformaldehyde (Sigma-Aldrich, Tokyo, Japan) for 30 min. The samples were next dehydrated sequentially in 10%, 15%, and 20% sucrose solutions, for 6 h in each solution. Next, the samples were embedded in the OCT compound (Sakura Finetek, Tokyo, Japan) and stored at −80°C. Each section was cut into 5-µm thickness by Leica CM1860 cryostat (Leica, Tokyo, Japan) and mounted on a glass slide. These sections were blocked with 5% bovine serum albumin (BSA; Iwai, Tokyo, Japan) for 30 min at room temperature. After three washes with 2% BSA, the samples were incubated with goat anti-rat PECAM-1 antibody (1:100; Millipore, Tokyo, Japan) for 2 h at room temperature. Following three PBS washes, PE-conjugated mouse anti-goat antibody (Jackson ImmunoResearch, West Grove, PA, USA) was used to cover the sample smears for 2 h at room temperature in dark. Following three PBS washes, the samples were added 4’6-diamidino-2-phenylindole-(DAPI)-DNA special packaging (ProLong, Tokyo, Japan). Fluorescent images were captured using an Axio Vert.A1 (ZEISS, Tokyo, Japan) fluorescent microscope.

We detected the apoptotic cells in the MI heart by terminal deoxynucleotidyl transferase dUTP nick end labeling (TUNEL) assay using in situ cell death detection kit, TMR red (Roche, Tokyo, Japan). The frozen section was washed by PBS two times and incubated in triton-X100 (Sigma-Aldrich, Tokyo, Japan) for 15 min on chamber slides. TUNEL reaction buffer was mixed and added on samples, which were then incubated in a humidified atmosphere for 60 min at 37°C in the dark. The samples were next analyzed under a fluorescence microscope, using an excitation wavelength of 570–620 nm and a detection wavelength of 345–455 nm. The apoptosis rate in whole section was calculated using NIH ImageJ software.

### Western blotting

The collected cells or the peri-infarct area of the heart was solubilized in PBS containing a protease inhibitor cocktail (Thermo Scientific, Yokohama, Japan), 1% sodium dodecyl sulfate, and 5 mM EDTA. The lysate was loaded into a 12% polyacrylamide gel, electrophoresed, and transferred to a polyvinylidene difluoride membrane. The membrane was incubated with primary antibodies (cleaved caspase-3, ANGPTL2; Cell-signaling Technology Japan, Tokyo, Japan; GAPDH, Sigma-Aldrich, Tokyo, Japan), and subsequently incubated with HRP-conjugated secondary antibodies (Jackson ImmunoResearch Laboratories, West Grove, PA, USA). The signal was detected using a chemiluminescence detection kit (Thermo Scientific, Yokohama, Japan). Densitometric analysis of the bands was done using NIH ImageJ software.

### Statistical analysis

All measurements are reported as mean ± standard deviation (SD). Comparison of all data was first performed using the one-way ANOVA function in the statistical product and service solutions software (SPSS, IBM Japan, Tokyo, Japan). Comparisons of the RT-PCR and ELISA results were followed by post-hoc tests using the Statcel 3 software (O.M.S. publisher, Saitama, Japan). Comparisons of mean heart function were performed using least-significant difference. P<0.05 was selected as the threshold for statistical significance.

## Results

### Sorting of outgrowth cells

**Figure 1:**
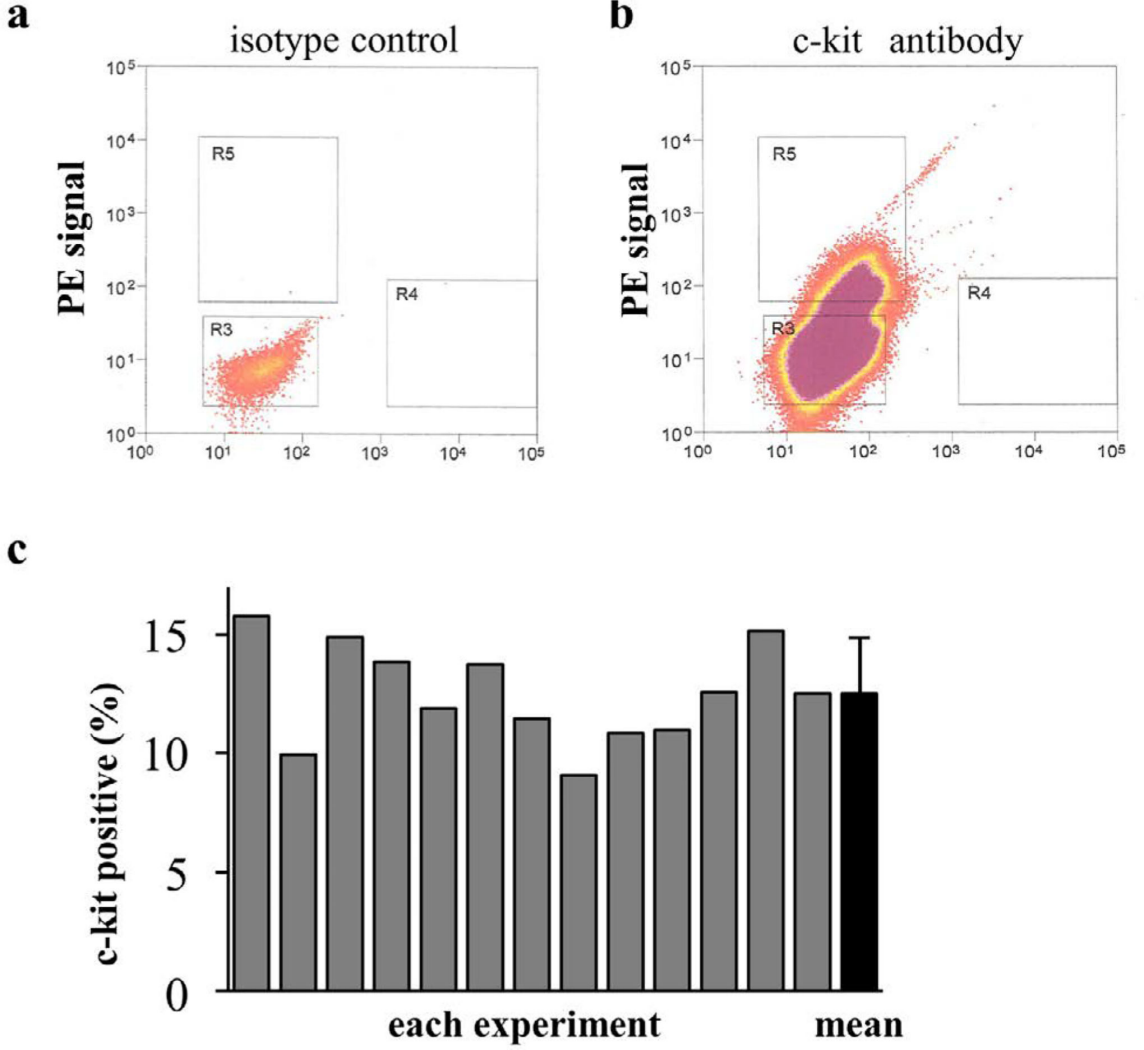
Percentage of c-kit+ cells in whole cell populations Flow cytometric sorting of c-kit+ cells using a phycoerythrin (PE)-conjugated anti-c-kit antibody. (a) An isotype-matched PE-conjugated antibody was used as a control. (b) Cells located inside the square “R5” were considered c-kit+ cells and those located inside the square “R3” were considered c-kit− cells. (c) The percentage of c-kit+ cells in each experiment ranged from 9.9% to 15.8%, and the mean value was 12.5 ± 2.1%.

Outgrowth cells were sorted into c-kit-positive and c-kit negative populations by FACS (Figure 1a and 1b). In total, for all of the experiments, sorted cells were prepared 13 times; cells were obtained from the same rat strain in each case. The percentage of c-kit+ cells in each experiment is shown in Figure 1c; the mean value of the percentage of c-kit+ cells was 12.5 ± 2.1%.

### c-kit+ Cells express more angiogenesis-related cytokines

**Figure 2:**
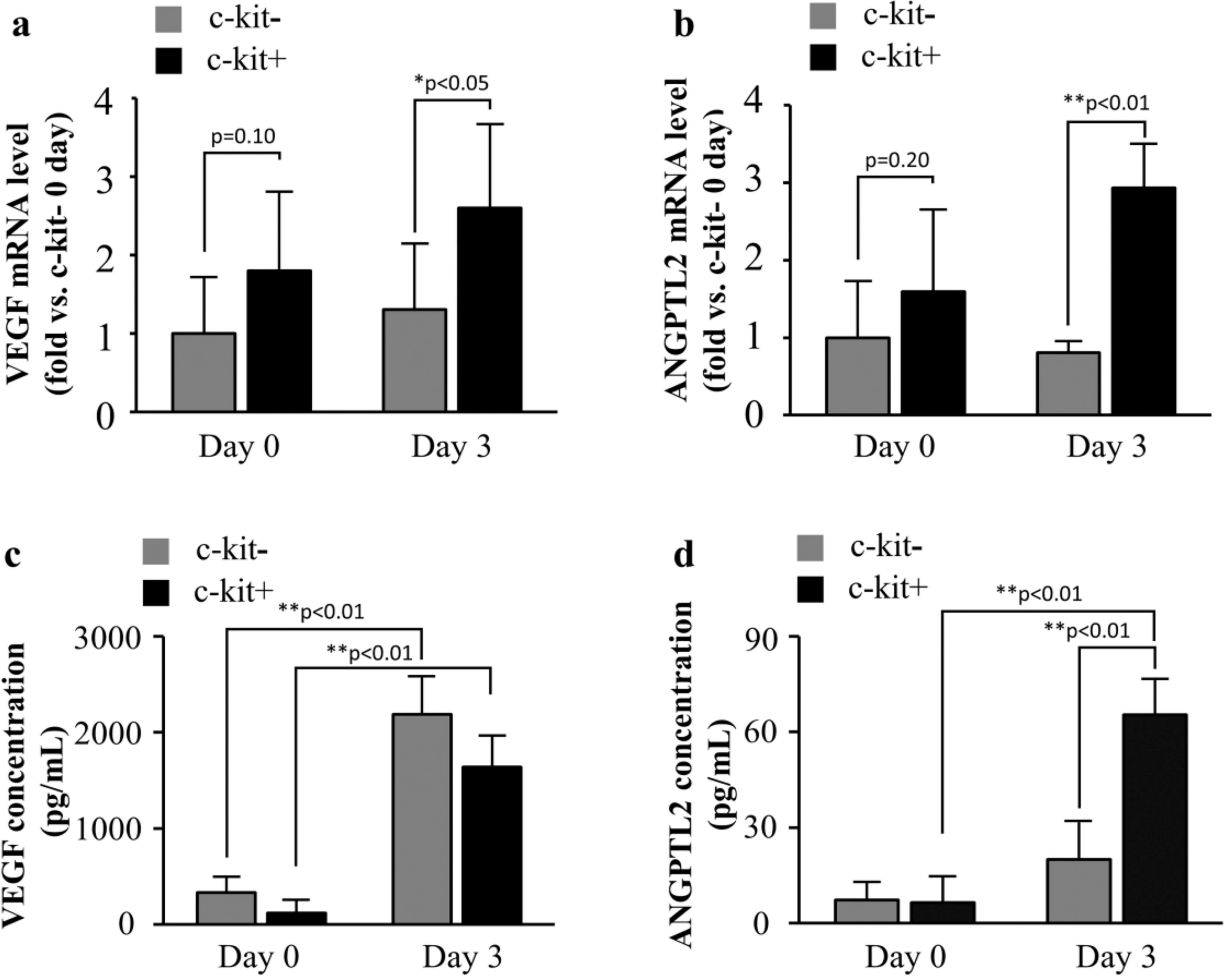
Expression and secretion of angiogenesis-related cytokines (a,b) The expression levels of cytokines were measured by RT-PCR. The expression of VEGF-mRNA and ANGPTL2-mRNA were no difference between the groups at day 0. After 3 d of hypoxia, the expression of both genes was significantly higher in the c-kit+ group. (c,d) The secretion levels of VEGF and ANGPTL2 were measured by ELISA. The secretion of these cytokines did not differ significantly between groups at day 0. VEGF secretion was significantly increased after 3 d of hypoxia in both groups. The secretion of ANGPTL2 also increased in both groups; however, only the c-kit+ increase exhibited statistical significance. In addition, the concentration of ANGPTL2 in the c-kit+ group was significantly higher than in the c-kit− group at day 3. (*p<0.05; **p<0.01)

The gene expression levels of angiogenesis-related cytokines (VEGF-mRNA and ANGPTL2-mRNA) are shown in Figure 2a and 2b. The expression of both genes at baseline (day 0) did not differ between the groups (VEGF: c-kit− vs. c-kit+=1.8 ± 1.0-fold; p=0.10, ANGPTL2: c-kit− vs. c-kit+=1.6 ± 1.0-fold; p=0.20). After 3 d of hypoxia (day 3), it was significantly higher in the c-kit+ group than in the c-kit− group (VEGF: c-kit−=1.3 ± 0.8-fold vs. c-kit+=2.6 ± 1.0-fold; p<0.05, ANGPTL2: c-kit−=0.8 ± 0.1-fold vs. c-kit+=2.9 ± 0.6-fold; p<0.01).

Next, the secreted cytokines in hypoxic culture medium were quantified by ELISA (Figure 2c and 2d). The secretion of VEGF was significantly increased at day 3 in both the c-kit− and the c-kit+ groups, compared to day 0 (c-kit−: day 0=330.1 ± 151.3 pg/mL vs. day 3=189.1 ± 380.0 pg/mL; p<0.01, c-kit+: day 0=118.1 ± 124.9 pg/mL vs. day 3=1636.5 ± 319.3 pg/mL; p<0.01); meanwhile, we found no statistical difference between the groups on day 0 or 3 (p=0.11 and p=0.70, respectively). Relative to the expression in ANGPTL2, the c-kit+ cells secreted more ANGPTL2 at day 3 than at day 0 (day 0=6.5 ± 7.9 pg/mL vs. day 3=65.5 ± 10.8 pg/mL; p<0.01). The secretion of ANGPTL2 in the c-kit− cells also tended to increase, although this increase did not reach statistical difference (day 0=7.3 ± 5.2 pg/mL vs. day 3=20.0 ± 11.6 pg/mL, p=0.93). Moreover, although the secretion of ANGPTL2 was not significantly different between the groups at day 0 (p=0.50), at day 3 this secretion was significantly higher in the c-kit+ cells than in the c-kit− cells (p<0.01).

### Anti-apoptotic property of c-kit+ cells

**Figure 3:**
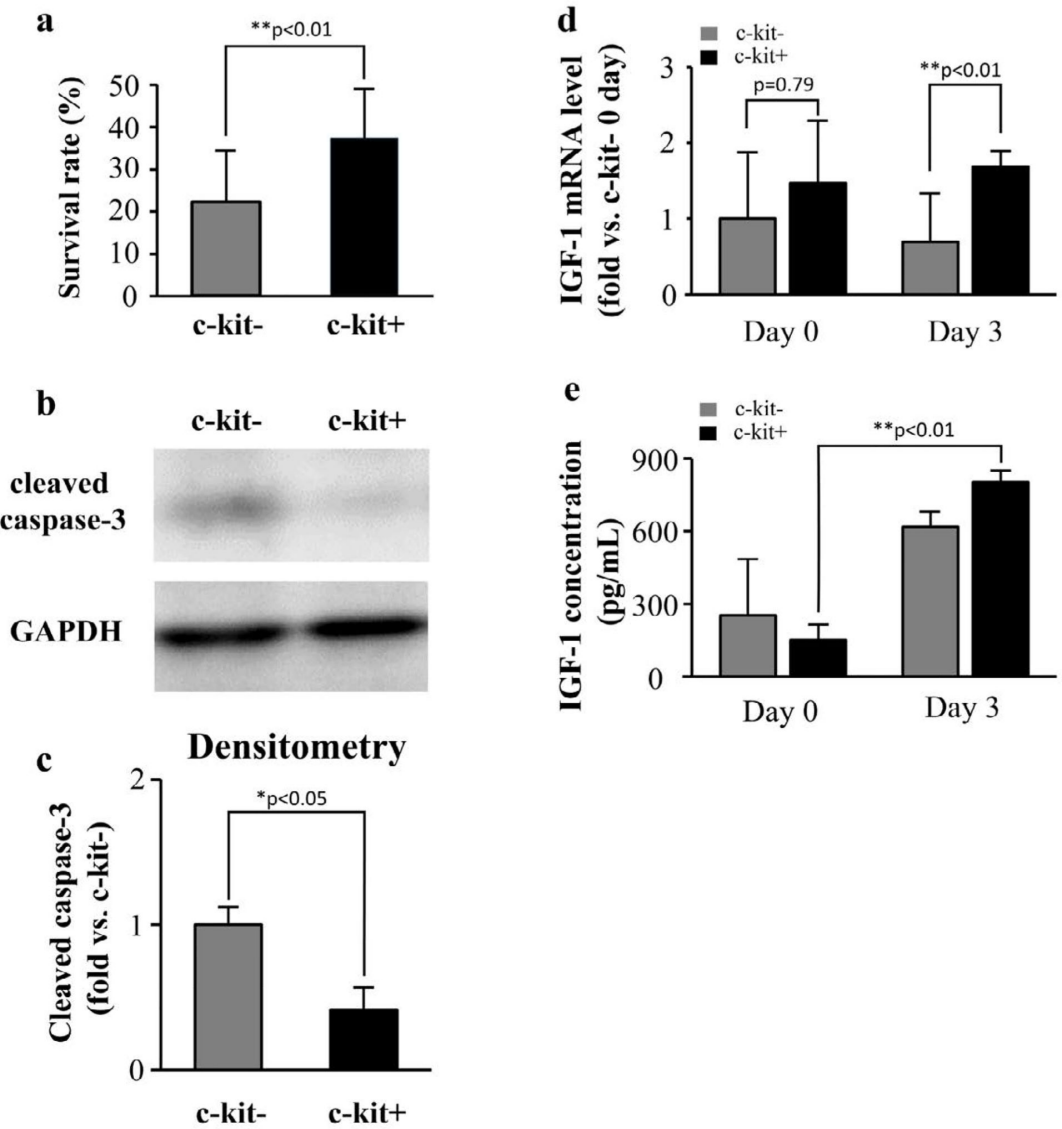
Anti-apoptosis effect and IGF-1 expression (a) The number of the remained cells in the c-kit+ group was significantly higher than that in the c-kit− group after 3 d of hypoxia. (b) The remained cells were assessed with western blot to detect the expression of cleaved caspase-3. (c) The densitometric analysis of western blot revealed that the expression of cleaved caspase-3 was significantly more in the c-kit− group. (d) The IGF-1-mRNA expressed more in c-kit+ cells and (e) the secretion of IGF-1 significantly increased in c-kit+ cells between day 0 and day 3 (*p<0.05; **p<0.01).

While the number of the cells were reduced in both groups by 3 d exposure to hypoxia, the reduction was significantly attenuated in the c-kit+ group, relative to the c-kit− group (c-kit−=37.3 ± 11.7%, c-kit+=22.1 ± 12.0%, p<0.01, ratio to day 0, Figure 3a). Western blot analysis demonstrated that the expression of cleaved caspase-3 was significantly higher in the c-kit− cells than in the c-kit+ cells at day 3 (Figure 3b and 3c, c-kit− vs. c-kit+=0.3 ± 0.2-fold; p<0.01), suggesting that more apoptosis occurred in the c-kit− group.

Next, we determined IGF-1 expression by RT-PCR and ELISA. The expression of IGF-1-mRNA was significantly elevated for the c-kit− group at day 3 (c-kit−=0.7 ± 0.6-fold vs. c-kit+=1.7 ± 0.2-fold; p<0.01, Figure 3d), although we found no such difference at day 0 (c-kit− vs. c-kit+=1.5 ± 0.8-fold; p=0.79). ELISA results also demonstrated that the secretion of IGF-1 did not differ between groups at day 0 (c-kit−=253.9 ± 228.2 pg/mL vs. c-kit+=152.0 ± 60.4 pg/mL; p=0.52); however, secretion in the c-kit+ group increased significantly after 3 d of hypoxia (c-kit−=619.3 ± 57.7 pg/mL vs. c-kit+=802.3 ± 44.9 pg/mL; p<0.01, Figure 3e). These results suggested that c-kit+ cells have anti-apoptotic property, which may be enhanced by hypoxic condition via IGF-1 cascade.

### Beneficial role of c-kit+ cells in the MI heart

**Figure 4:**
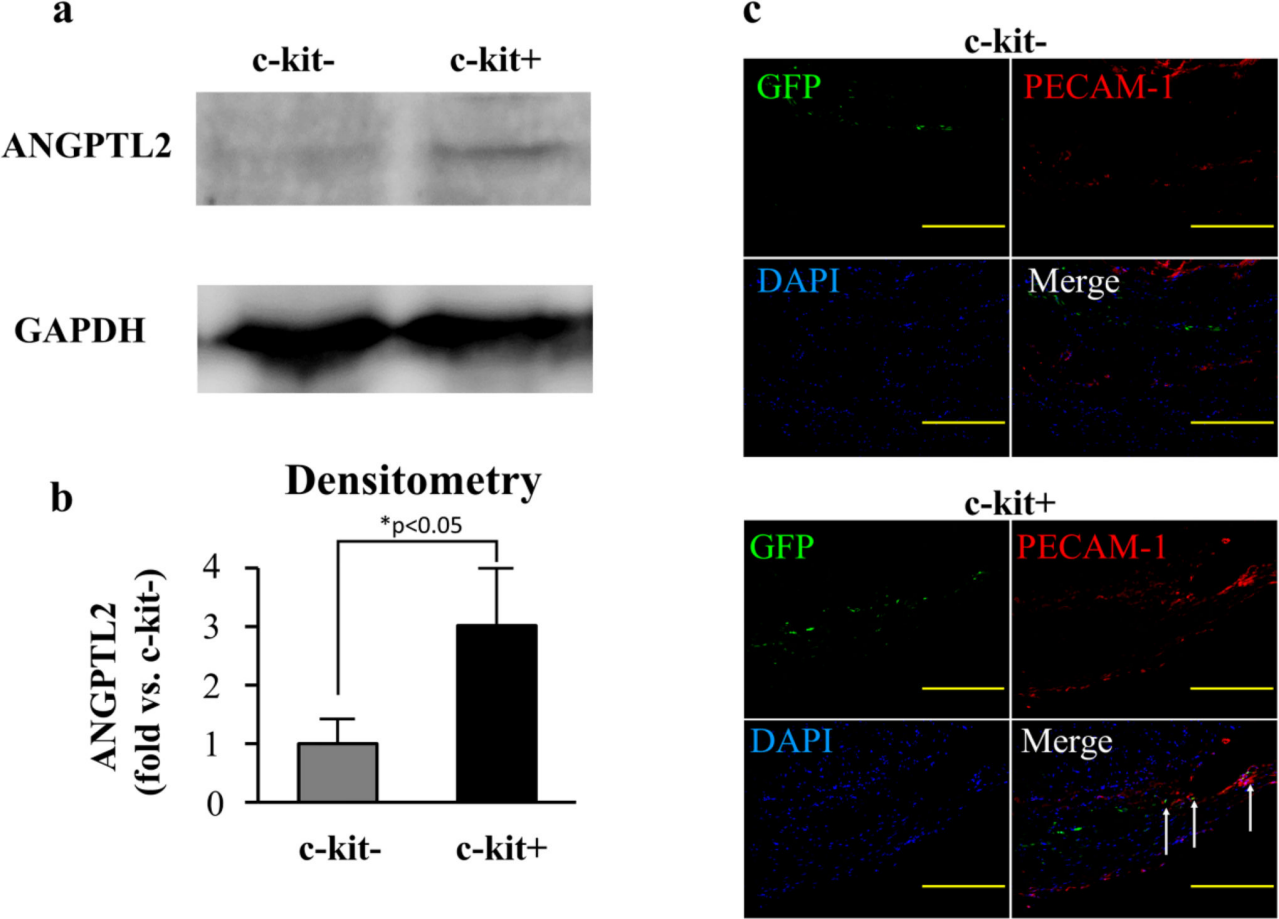
Angiogenesis ability of c-kit+ cells in MI heart (a) The MI heart was accessed with western blot to detect the ANGPTL2 expression. (b) The densitometric analysis demonstrated that ANGTPL2 expression was significantly elevated in the c-kit+ group at 4 weeks after treatment. (c) The heart sections were stained for PECAM-1 (red signal) and nuclei (blue signal). In the c-kit+ group, the PECAM-1 signals co-located with injected cells (GFP positive cells; green signal) in some areas. (*p<0.05, Scale bar=200 µm)

At 4 weeks after the MI induction, western blot analysis demonstrated that the expression of ANGPTL2 was elevated in the hearts injected with c-kit+ cells (c-kit− vs. c-kit+=2.9 ± 0.8-fold; p<0.05, Figure 4a). Furthermore, immunostaining of the peri-infarct area from the 4-week hearts revealed that PECAM-1 signals were elevated in the c-kit+ group. In addition, the PECAM-1 signal was colocalized with injected cells (GFP+ cells, Figure 4b).

**Figure 5:**
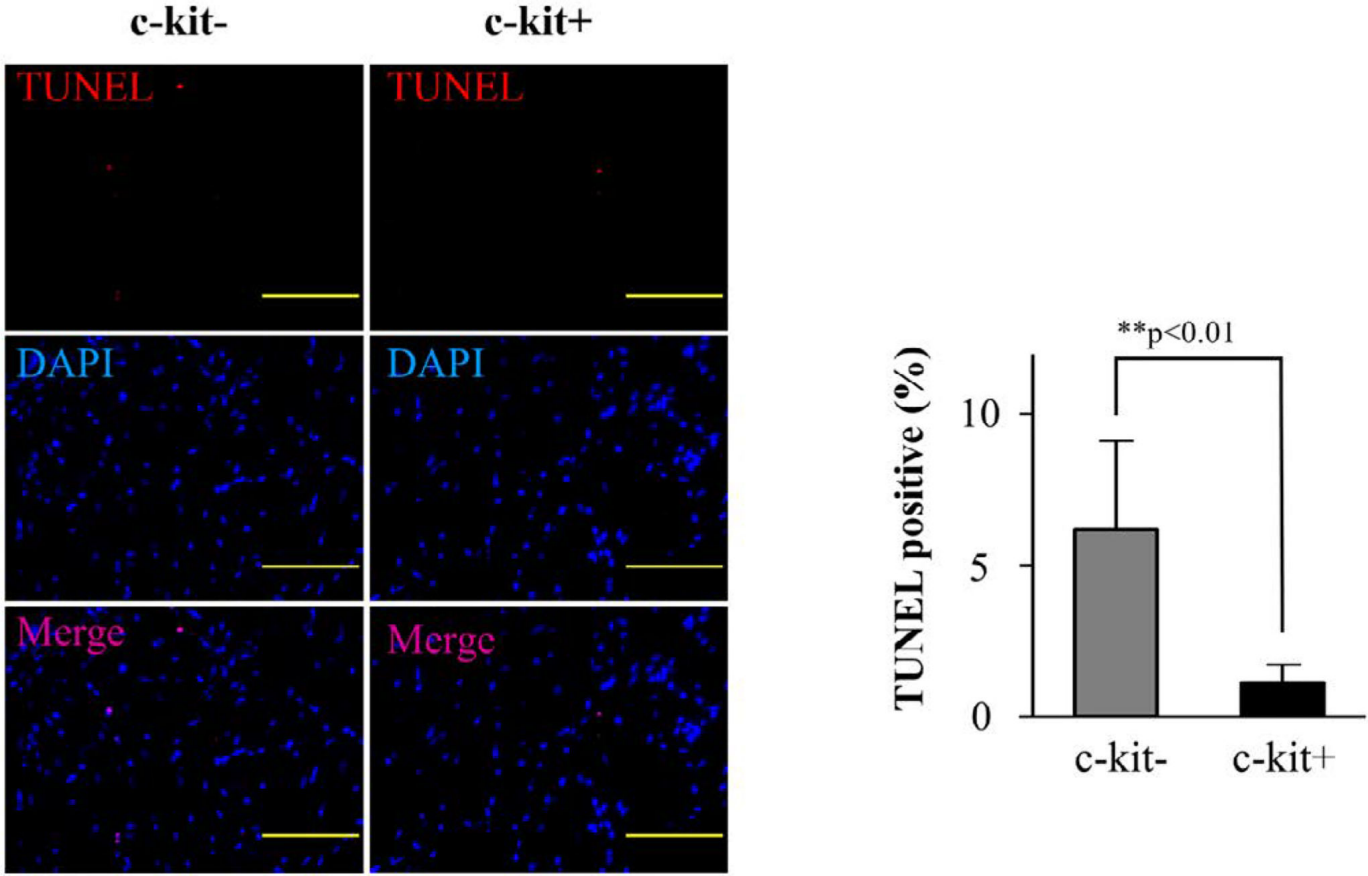
Detection of apoptosis in MI heart TUNEL staining was performed in the MI heart at 4 weeks after treatment. Quantitative analysis of the TUNEL positive cells per nuclei in peri-infarct area represented that the c-kit+ group contained significant less apoptotic cells than the c-kit− group. (**p<0.01, Scale bar=100 µm)

To evaluate the anti-apoptotic effect of c-kit+, TUNEL staining was performed. Semi-quantitative analysis of TUNEL-positive cells indicated that the hearts injected with c-kit+ cells expressed weaker TUNEL-positive signals than the hearts injected with c-kit− cells (c-kit−=6.2 ± 2.9% vs. c-kit+=1.1 ± 0.6%; p<0.01, Figure 5).

**Figure 6:**
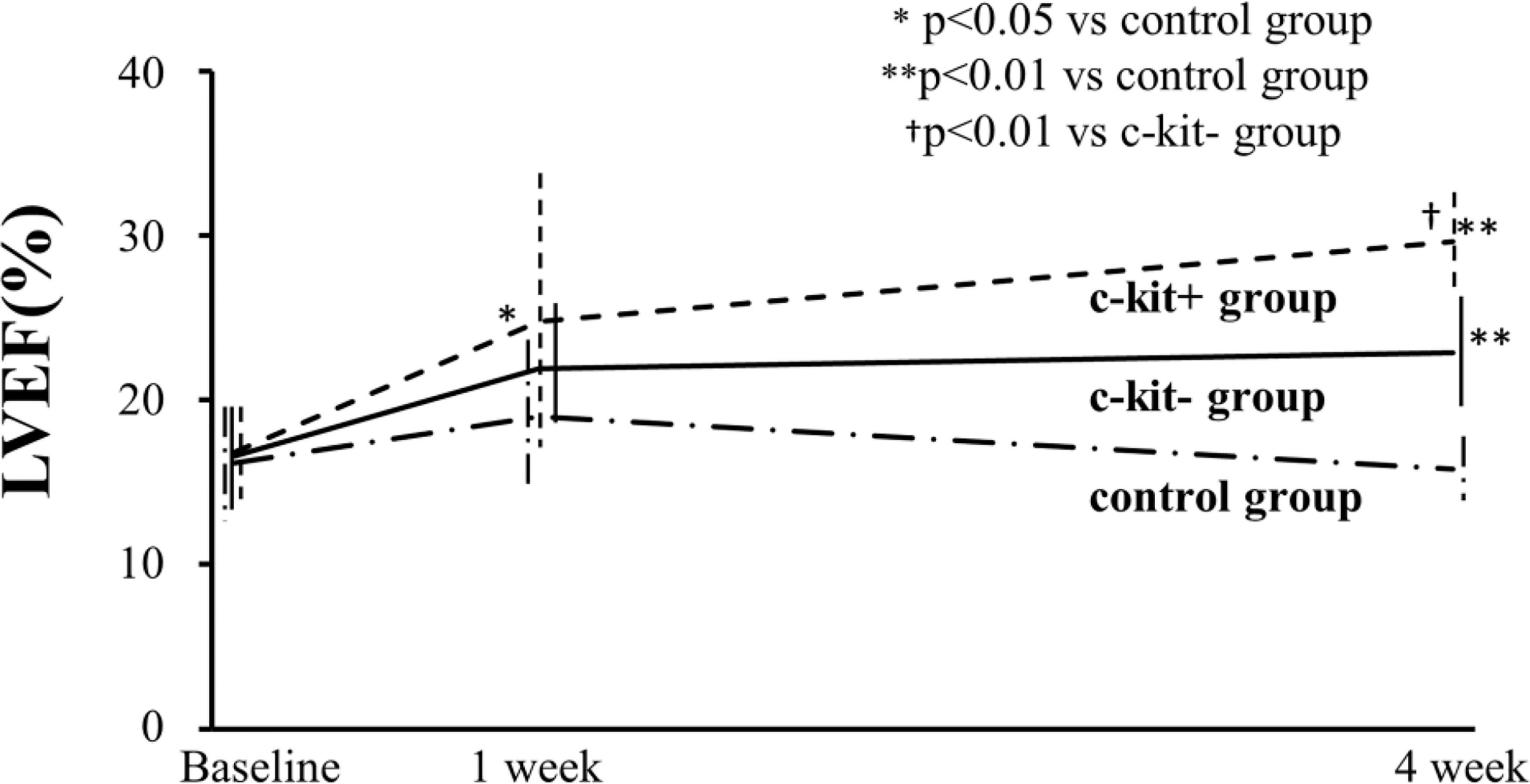
Cardiac function of MI hearts over time Time course of cardiac function changes, in terms of LVEF. The baseline LVEF for the three groups (the day after surgery) did not significantly differ. After 1 week, the LVEF of the c-kit+ group was significantly higher than that of the control group, although differences were not significant between control and c-kit, or between c-kit− and c-kit+. At 4 weeks after the surgery, the LVEF of c-kit+ is significant higher than the control and c-kit− group. The LVEF of c-kit− group also show significant higher than the control group.

Figure 6 shows the time course change of the LVEF for up to 4 weeks following treatment. At baseline (immediately after surgery), LVEF did not differ between the control (hydrogel only), c-kit−, and c-kit+ groups (control=16.2 ± 3.0%, c-kit−=16.6 ± 3.4%, c-kit+=16.8 ± 2.9%; p=0.86 in control vs. c-kit−; p=0.78 in control vs. c-kit+; and p = 0.81 in c-kit− vs. c-kit+). One week after treatment, the LVEF in the c-kit+ group was significantly higher than in the control group (control=19.0 ± 4.5% vs. c-kit+=24.8 ± 8.2%; p<0.05), although we found no significant difference between the control and c-kit− groups (c-kit−=21.9 ± 4.1%, p=0.83), or between the c-kit− and c-kit+ groups (p=0.92). After 4 weeks, the LVEF in the c-kit+ group was significantly higher than in the c-kit− group and the control group (control=15.8 ± 3.4%, c-kit− = 22.9 ± 3.2%, and c-kit+ = 29.7 ± 2.3%; p<0.01 in control vs. c-kit−, p<0.01 in control vs. c-kit+, and p<0.01 in c-kit− vs. c-kit+).

## Discussion

### Higher ability of angiogenesis in c-kit positive cells

In the current study, we directly compared cells grouped by their c-kit status to assess the potential relevance of this marker for cell therapy.

It has been shown that c-kit positive cardiac stem cells can differentiate into heart-forming cells, including vessel and endothelial cells [8,10]. Our *in vitro* experiments demonstrated that there were no differences in angiogenesis ability between c-kit+ and c-kit− cardiac outgrowth cells under normal conditions (normoxia); VEGF and ANGPTL2 gene expression and cytokine secretion tests confirmed this result. When exposed to hypoxic stress, both cell types showed angiogenesis effects, and there was no statistically significant difference in cytokines expression after hypoxia for 3 days. However, in the hearts harvested at 4 weeks after treatment, ANGPTL2 expression was found to be higher in c-kit+ cell-injected hearts. Furthermore, immunostaining of the heart section after 4 weeks also revealed that signals for the endothelial cell marker PECAM-1 were elevated in the c-kit+ group. Notably, a portion of injected (GFP positive) cells was colocalized with PECAM-1 signals in the c-kit+ cell-administered heart, although this effect was barely visible in the heart administered c-kit− cells. In addition, the increase in heart function was greater in c-kit+ cells than in c-kit− cells. ANGPTL2 is known to regulate sprouting angiogenesis, and can control the adaptive vascular endothelium [22,23]. These results suggest that c-kit+ cells, but rarely c-kit− cells, can directly differentiate into endothelial cells. The further improvement of LVEF at 4 weeks after treatment in c-kit+ cells over c-kit− cells may be related to the higher secretion of cytokines and direct differentiation effects.

### Anti-apoptotic ability of c-kit positive cells

It has been reported that the expression of c-kit+ cardiac stem cells was increased when the heart was exposed to a stress, e.g., myocardial infarction, heart failure, or after exercise in animal [24,25] and human [26] heart. In normal condition, the oxygen concentration of a heart tissue is around 5% [27]. When a heart is in moderate hypoxia, it drops to less than 3% [28]. Previous reports suggest that cell culture in 5% of oxygen concentration preserves the proliferation ability in hematopoietic stem cells [29], or in c-kit+ cardiac stem cells [25]. In our experiments, the oxygen concentration was set to 3%, to simulate hypoxic conditions. As a result, while the number of the cells was decreased in both groups after 3 d of hypoxic culture, the number of the cells was significantly higher in the c-kit+ group. The expression of cleaved-caspase 3, an apoptosis marker [30], was significantly lower in c-kit+ cells, and the expression of IGF-1, which is known to play an important role in proliferation and repairing damaged tissue by inhibiting apoptosis [31,32], was higher in c-kit+ cells. Consistently, our *in vivo* experiments demonstrated that the TUNEL positive cells were significantly lower in c-kit+. In addition, the expression of Ki-67, a marker for cell proliferation, did not differ between the groups *in vitro* (data not shown). These results suggest that c-kit+ cells have a greater anti-apoptosis ability than c-kit− cells, although our data do not confirm whether c-kit+ cells can proliferate faster than c-kit− cells. The higher retention rate confirmed *in vivo* and *in vitro* may imply further improvement of cardiac function by c-kit+ cells (Figure 6).

### Cell administration with hydrogel

The cells that we administered into the MI heart were conjugated with hydrogel to prolong their effects. The gel used in this study was thermosensitive, biodegradable, and stiffness-controllable [19,20]. When a saline solution is used as a vehicle for an intra-coronary or intra-muscular injection of these cells, the cell retention rate is relatively low [25,33]. In contrast, administration of the cells in the hydrogel markedly increases the retention rate, and increases IGF-1 secretion, improving functional recovery [19]. Since our gel lasts ~ 4 weeks after injection, the injected (GFP positive) cells remained in the heart when we harvested it at the 4-week time point. It is known that at the early phase of MI, acute inflammation is induced. Such a circumstance is highly harmful to the cells, which may reduce the differentiation and attenuate the benefit of cell therapy [34]. A previous report suggested that an inflammatory environment at 3–7 d may have influenced the effectiveness of heart regeneration [28]. Therefore, acute preservation of myocardium around 3–6 d after occurrence of MI provides the benefit of the cell therapy [28]. In addition, it took 10–28 d for injected stem cells to attach to the recipient heart and differentiate into mature cells [8,35]. To maximize the therapeutic benefit, cell administration should be performed using duration-controllable material, such as hydrogel, or using a cell sheet technique.

### Potential epigenetic mechanism of c-kit cells

Recent study has found histone methyltransferase G9a is required for cardiomyocyte homeostasis and hypertrophy [36]. The main epigenetic function of G9a is establishing H3K9me2 [37] as well as maintenance of DNA methylation at certain loci [38]. Apart from being as an epigenetic modifier, G9a also plays a role in the regulation of signalling. For instance, G9a has been found to negatively regulate MAPK signalling pathway [39]. As c-Kit promotes growth and migration of human cardiac progenitor cells via regulating of the PI3K/AKT and MAPK signalling pathways [40–43], G9a seems to exert an antagonistic function of c-Kit in the controlling of MAPK signalling pathway. Therefore, investigation the conflicts of c-Kit as well as histone modifier G9a in the regulation of signalling pathway might better understand the potential regulatory machinery involved in cardiac stem cells and facilitate the therapeutic function of these cells in the clinical therapy.

## Conclusions

Our study demonstrated that both c-kit− and c-kit+ cell treatment following MI improved heart function. In addition, c-kit+ cells showed higher angiogenesis and anti-apoptotic effects than c-kit− cells, resulting in better recovery in heart function.

## References

[1] Go AS, Mozaffarian D, Roger VL, Benjamin EJ, Berry JD (2013). Heart disease and stroke statistics-2013 update: a report from the American Heart Association. Circulation.

[2] Khan AR, Farid TA, Pathan A, Tripathi A, Ghafghazi S (2016). Impact of Cell Therapy on Myocardial Perfusion and Cardiovascular Outcomes in Patients With Angina Refractory to Medical Therapy: A Systematic Review and Meta-Analysis. Circ Res.

[3] Pfister O, Della Verde G, Liao R, Kuster GM (2014). Regenerative therapy for cardiovascular disease. Transl Res.

[4] Messina E, De Angelis L, Frati G, Morrone S, Chimenti S (2004). Isolation and expansion of adult cardiac stem cells from human and murine heart. Circ Res.

[5] Matsuura K, Nagai T, Nishigaki N, Oyama T, Nishi J (2004). Adult cardiac Sca-1-positive cells differentiate into beating cardiomyocytes. J Biol Chem.

[6] Moretti A, Caron L, Nakano A, Lam JT, Bernshausen A (2006). Multipotent embryonic isl1+ progenitor cells lead to cardiac, smooth muscle, and endothelial cell diversification. Cell.

[7] Iancu CB, Iancu D, Renţea I, Hostiuc S, Dermengiu D (2015). Molecular signatures of cardiac stem cells. Rom J Morphol Embryol.

[8] Beltrami AP, Barlucchi L, Torella D, Baker M, Limana F (2003). Adult cardiac stem cells are multipotent and support myocardial regeneration. Cell.

[9] Linke A, Müller P, Nurzynska D, Casarsa C, Torella D (2005). Stem cells in the dog heart are self-renewing, clonogenic, and multipotent and regenerate infarcted myocardium, improving cardiac function. Proc Natl Acad Sci U S A.

[10] Dawn B, Stein AB, Urbanek K, Rota M, Whang B (2005). Cardiac stem cells delivered intravascularly traverse the vessel barrier, regenerate infarcted myocardium, and improve cardiac function. Proc Natl Acad Sci U S A.

[11] Tang XL, Rokosh G, Sanganalmath SK, Yuan F, Sato H (2010). Intracoronary administration of cardiac progenitor cells alleviates left ventricular dysfunction in rats with a 30-day-old infarction. Circulation.

[12] Fazel S, Cimini M, Chen L, Li S, Angoulvant D (2006). Cardioprotective c-kit+ cells are from the bone marrow and regulate the myocardial balance of angiogenic cytokines. J Clin Invest.

[13] Bolli R, Chugh AR, D'Amario D, Loughran JH, Stoddard MF (2011). Cardiac stem cells in patients with ischaemic cardiomyopathy (SCIPIO): initial results of a randomised phase 1 trial. Lancet.

[14] Sawa Y (2007). Surgical regeneration therapy using myoblast sheets for severe heart failure. Kyobu Geka.

[15] Kobayashi H, Shimizu T, Yamato M, Tono K, Masuda H (2008). Fibroblast sheets co-cultured with endothelial progenitor cells improve cardiac function of infarcted hearts. J Artif Organs.

[16] Makkar RR, Smith RR, Cheng K, Malliaras K, Thomson LE (2012). Intracoronary cardiosphere-derived cells for heart regeneration after myocardial infarction (CADUCEUS): a prospective, randomised phase 1 trial. Lancet.

[17] Davis DR, Zhang Y, Smith RR, Cheng K, Terrovitis J (2009). Validation of the cardiosphere method to culture cardiac progenitor cells from myocardial tissue. PLoS One.

[18] van Berlo JH, Kanisicak O, Maillet M, Vagnozzi RJ, Karch J (2014). c-kit+ cells minimally contribute cardiomyocytes to the heart. Nature.

[19] Matsushita S, Forrester JS, Li C, Sato M, Li Z (2016). Administration of cells with thermosensitive hydrogel enhances the functional recovery in ischemic rat heart. J Tissue Eng.

[20] Li Z, Guo X, Matsushita S, Guan J (2011). Differentiation of cardiosphere-derived cells into a mature cardiac lineage using biodegradable poly (N-isopropylacrylamide) hydrogels. Biomaterials.

[21] Matsushita S, Naito M, Amano A (2014). Micro-computed tomography provides accurate measurement for cardiac function in infarcted rat heart. Open J Med Imag.

[22] Maisonpierre PC, Suri C, Jones PF, Bartunkova S, Wiegand SJ (1997). Angiopoietin-2, a natural antagonist for Tie2 that disrupts in vivo angiogenesis. Science.

[23] Stratmann A, Risau W, Plate KH (1998). Cell type-specific expression of angiopoietin-1 and angiopoietin-2 suggests a role in glioblastoma angiogenesis. Am J Pathol.

[24] Fransioli J, Bailey B, Gude NA, Cottage CT, Muraski JA (2008). Evolution of the c-kit-positive cell response to pathological challenge in the myocardium. Stem Cells.

[25] Lee ST, White AJ, Matsushita S, Malliaras K, Steenbergen C (2011). Intramyocardial injection of autologous cardiospheres or cardiosphere-derived cells preserves function and minimizes adverse ventricular remodeling in pigs with heart failure post-myocardial infarction. J Am Coll Cardiol.

[26] Itzhaki-Alfia A, Leor J, Raanani E, Sternik L, Spiegelstein D (2009). Patient characteristics and cell source determine the number of isolated human cardiac progenitor cells. Circulation.

[27] Roy S, Khanna S, Wallace WA, Lappalainen J, Rink C (2003). Characterization of perceived hyperoxia in isolated primary cardiac fibroblasts and in the reoxygenated heart. J Biol Chem.

[28] Forrester JS, White AJ, Matsushita S, Chakravarty T, Makkar RR (2009). New paradigms of myocardial regeneration post-infarction: tissue preservation, cell environment, and pluripotent cell sources. JACC Cardiovasc Interv.

[29] Cipolleschi MG, Dello Sbarba P, Olivotto M (1993). The role of hypoxia in the maintenance of hematopoietic stem cells. Blood.

[30] Ferrer I, Planas AM (2003). Signaling of cell death and cell survival following focal cerebral ischemia: life and death struggle in the penumbra. J Neuropathol Exp Neurol.

[31] Niu XL, Li J, Hakim ZS, Rojas M, Runge MS (2007). Leukocyte antigen-related deficiency enhances insulin-like growth factor-1 signaling in vascular smooth muscle cells and promotes neointima formation in response to vascular injury. J Biol Chem.

[32] Patel VA, Zhang QJ, Siddle K, Soos MA, Goddard M (2001). Defect in insulin-like growth factor-1 survival mechanism in atherosclerotic plaque-derived vascular smooth muscle cells is mediated by reduced surface binding and signaling. Circ Res.

[33] Hou D, Youssef EA, Brinton TJ, Zhang P, Rogers P (2005). Radiolabeled cell distribution after intramyocardial, intracoronary, and interstitial retrograde coronary venous delivery: implications for current clinical trials. Circulation.

[34] Li X, Tamama K, Xie X, Guan J (2016). Improving Cell Engraftment in Cardiac Stem Cell Therapy. Stem Cells Int.

[35] Bolli R, Tang XL, Sanganalmath SK, Rimoldi O, Mosna F (2013). Intracoronary delivery of autologous cardiac stem cells improves cardiac function in a porcine model of chronic ischemic cardiomyopathy. Circulation.

[36] Papait R, Serio S, Pagiatakis C, Rusconi F, Carullo P (2017). Histone Methyltransferase G9a Is Required for Cardiomyocyte Homeostasis and Hypertrophy. Circulation.

[37] Tachibana M, Sugimoto K, Fukushima T, Shinkai Y (2001). Set domain-containing protein, G9a, is a novel lysine-preferring mammalian histone methyltransferase with hyperactivity and specific selectivity to lysines 9 and 27 of histone H3. Journal of Biological Chemistry.

[38] Zhang T, Termanis A, Özkan B, Bao XX, Culley J (2016). G9a/GLP complex maintains imprinted DNA methylation in embryonic stem cells. Cell reports.

[39] Hua KT, Wang MY, Chen MW, Wei LH, Chen CK (2014). The H3K9 methyltransferase G9a is a marker of aggressive ovarian cancer that promotes peritoneal metastasis. Molecular cancer.

[40] Vajravelu BN, Hong KU, Al-Maqtari T, Cao P, Keith MC (2015). C-Kit promotes growth and migration of human cardiac progenitor cells via the PI3K-AKT and MEK-ERK pathways. PloS one.

[41] Siaghy EM, Devaux Y, Sfaksi N, Carteaux JP, Ungureanu-Longrois D (2000). Consequences of inspired oxygen fraction manipulation on myocardial oxygen pressure, adenosine and lactate concentrations: a combined myocardial microdialysis and sensitive oxygen electrode study in pigs. J Mol Cell Cardiol.

[42] He JQ, Vu DM, Hunt G, Chugh A, Bhatnagar A (2011). Human cardiac stem cells isolated from atrial appendages stably express c-kit. PLoS One.

[43] Keith MC, Bolli R (2015). "String theory" of c-kit(pos) cardiac cells: a new paradigm regarding the nature of these cells that may reconcile apparently discrepant results. Circ Res.

